# Antimony Resistance in *Leishmania*, Focusing on Experimental Research

**DOI:** 10.1155/2011/695382

**Published:** 2011-11-17

**Authors:** Fakhri Jeddi, Renaud Piarroux, Charles Mary

**Affiliations:** Laboratoire de Parasitologie, Hôpital de la Timone, 264 rue Saint-Pierre, 13385 Marseille Cedex 05, France

## Abstract

Leishmaniases are parasitic diseases that spread in many countries with a prevalence of 12 million cases. There are few available treatments and antimonials are still of major importance in the therapeutic strategies used in most endemic regions. However, resistance toward these compounds has recently emerged in areas where the replacement of these drugs is mainly limited by the cost of alternative molecules. In this paper, we reviewed the studies carried out on antimonial resistance in *Leishmania*. Several common limitations of these works are presented before prevalent approaches to evidence antimonial resistance are related. Afterwards, phenotypic determination of resistance is described, then confronted to clinical outcome. Finally, we detail molecular mechanisms and targets involved in resistance and already identified *in vitro* within selected mutant strains or in clinical isolates.

## 1. Introduction

Leishmaniases are a set of vector-borne diseases caused by a flagellate protozoan transmitted by the bite of an insect vector, the phlebotomine sandfly. This group of diseases affects 98 countries with three distinct entities: cutaneous, mucocutaneous, and visceral leishmaniasis [1]. Various clinical outcomes are described from a simple skin lesion that may heal spontaneously to a multi-organ failure, fatal if untreated. There is still no human vaccine against this disease [2] and therapy takes a major place in the control strategies. Moreover, there are few drugs available and their effectiveness is variable. The use of the liposomal form of amphotericin B, a highly active molecule with reduced side effects, is still restricted to the treatment of visceral leishmaniasis in countries that can afford its cost, such as European countries. However Europe accounts for less than 1% of the approximately 500,000 cases per year which occur mainly in the Indian subcontinent, Sudan, and Brazil [3]. Other molecules such as pentamidine, miltefosine, or fluconazole are available but their use is restricted owing to side effects, cost, or effectiveness [4]. This accounts for the still predominant place of antimony derivatives, sodium stibiogluconate (Pentostam), and meglumine antimoniate (Glucantime) which have been used in the treatment of the majority of cases of leishmaniases for more than 60 years worldwide. Currently, these molecules have two major limitations: first, side effects are frequent and can be serious; second, parasite resistance is emerging in some endemic areas, causing an increase in treatment failure [5, 6]. Resistance to antimonials has emerged over 20 years in the region of Bihar in India. Low dosage or insufficient duration of treatment led to the selection of resistant mutants that were transmitted more easily in this anthroponotic form than in areas where anthropozoonotic cycle occurs. Currently, in the most heavily affected areas of India, resistance to antimonials may reach more than 60% of the cases, thus representing a public health problem [7]. It imposed the use of other drugs such as amphotericine B or, more recently, miltefosine. Other endemic areas such as Sudan are also experiencing this phenomenon [8]. Cases of resistance have also been described in Europe for over a decade [9]. Here the selection occurs in dogs which, unlike men, are still being treated with antimonials [10]. In other mediterranean areas such as Maghreb, Albania, and Middle East except Israel, most infected dogs are left untreated; antimonials are still the first line treatment and display high efficiency [11, 12]. The issue of antileishmanial therapy depends on several factors such as the immune status of the host, the molecule, its preparation, its administration, and the susceptibility of the strain to the drug [13, 14].

Drug resistance of *Leishmania* may be natural, acquired when the parasite is exposed to suboptimal doses of the drug, or induced *in vitro* after selection of mutants by exposure to gradually increasing concentrations of the drug.

This review deals with molecular mechanisms in *Leishmania* resistance to antimonials. First, we relate methodology, general limitations, and tools used for resistance surveys. In a second step, we describe phenotypic determination of resistance and its correlation with clinical outcome. Finally, we focus on molecular resistance pathways and already identified targets within *in vitro* selected mutants and field isolates.

## 2. Methodologic Aspects

Several approaches have been used to study antimony resistance at cellular or molecular scales. They address the phenotypic, genomic, or proteomic levels. Before discussing the different ways of investigation, we present some general considerations related to *Leishmania* isolates, the evolution of their characteristics during maintenance *in vitro*, selection of mutants, and postulates about cross-resistance mechanisms.

### 2.1. General Limitations

#### 2.1.1. Parasites

**(1)* Leishmania* Species**

Several *in vitro* experiments were performed on *L. tarentolae,* a non-infective species for laboratory personnel [15–20]. By using this species, different resistance mechanisms and molecular targets were identified and their presence confirmed in other species selected *in vitro* or from clinical isolates. However, intrinsic susceptibility to heavy metals and thiols levels found in *L. tarentolae* can be very different from those encountered in *Leishmania* species of clinical interest [21].

**(2) Parasitic Stage and Resistance**

Most of *in vitro* resistance experiments are performed on promastigotes, easier to cultivate and to quantify than amastigotes. However, they do not represent the parasitic stage on which antimonials act *in vivo*. Promastigotes are usually not sensitive to SbV which has a stage-specific activity unless they are exposed to Pentostam^R^ and several experiments indicate that this susceptibility is not due to sodium stibiogluconate but rather to chlorocresol included in Pentostam formulation [22, 23]. Ephros et al. showed that *L. donovani* axenic amastigotes were considerably more susceptible to Pentostam than promastigotes. The two stages were also tested for chlorocresol susceptibility displaying almost the same IC50. Interestingly, chlorocresol concentration for Pentostam IC50 was eight-fold lower than chlorocresol IC50 in axenic amastigotes. Thus increased susceptibility of axenic amastigotes to Pentostam is mainly due to SbV [24]. In another study, *L. donovani* amastigotes were either SbIII or SbV (Glucantime or Pentostam chlorocresol free) sensitive unlike promastigotes which displayed a very high IC50. Susceptibility to SbV follows stage transformation from promastigotes to axenic amastigotes while resistance to SbV is acquired while amastigotes differentiate into promastigotes [25]. Promastigotes also display different and mostly higher thiols levels than amastigotes [26]. Carriers belonging to the ABC system and involved in the efflux or sequestration of antimonials have indeed been identified in promastigotes resistant mutants and few studies on the role of these proteins have been conducted on amastigotes.

**(3) Selection during Treatment of the Host**

The *in vitro* sensitivity of strains isolated from untreated hosts did not change despite the successive passages in culture or after animal infection. However, the sensitivity was reduced in strains isolated from either patients or dogs treated with antimonials [9, 10, 27]. The growth and proliferation of cultured strains isolated after treatment were lowered compared to the strains isolated before treatment [27].

#### 2.1.2. Conditions of Culture and Selection *In Vitro*

**(1) Culture Medium**

The growth rate of *L. infantum* promastigotes influences their IC50 for SbV chlorocresol-free formulations as reported by Carrio et al. In fact, different IC50 values can be obtained for the same strain by varying heat-inactivated fetal calf serum concentration in culture medium. Variation of pH can also influence promastigotes susceptibility to SbV mostly for Schneider's medium but also for M199 and RPMI media [28].

**(2) Impact of In Vitro Culture on Primary Phenotype**

*Leishmania* parasites isolated from an infected host may present a polyclonal population with a varied expression of different phenotypes such as growth capacity *in vitro*, infectivity, and drug resistance [29, 30]. Obtaining a sufficient number of promastigotes to conduct experiments often requires numerous passages and thus can induce changes in the expression pattern of the parasite. The possible selection of one or some clones that were not dominant at the time of parasite isolation may complicate the interpretation of a resistance survey. Indeed, the virulence of a strain and its capacity for growth and adaptation in culture can alter the clonal composition of strain over successive passages in the medium and so modify the expression of resistance phenotype. However, experiments have shown that during induction of *in vitro* resistance by discontinuous exposition of parasites to the drug, the resistance phenotype does not vary either during successive passages in culture in the absence of the drug or after *in vivo* infection [29, 30]. In addition, Antimonial resistant *L. donovani* Indian strains cultured with several passages before infection of macrophages or hamsters maintained their resistance profile [31]. Molecular markers analysis directly performed from biological samples should avoid this possible bias.

**(3) Use of Arsenicals for *In Vitro* Selection**

Several experiments on *Leishmania* resistance were performed with a progressive selection of mutant strains by exposing promastigotes to increasing concentrations of arsenicals [17, 20, 32, 33]. These studies suffer the selection bias of arsenic derivatives not used in antileishmanial therapy but whose use *in vitro* showed that they confer cross-resistance to antimonials. Arsenicals have undubitably common characteristics with antimonials in their mode of action such as increased reactive oxygen species (ROS), loss of mitochondrial membrane potential and collapse of the available quantity of ATP followed by a cell death that occurs by cell shrinkage and DNA fragmentation. The two related heavy metals require iron for their cytotoxic effect. However, arsenicals induce an increase in intracellular calcium that does not occur with antimonials. Conversely, the level of intracellular glutathione which is not affected during treatment with arsenicals is lowered when antimonials are used. The inhibition of calcium influx in case of arsenicals and glutathione supplementation in presence of antimony decreased cell death in both cases [34]. It is therefore conceivable that the mutant strains selected *in vitro* against arsenic derivatives and then tested against antimonials may have developed resistance mechanisms specific to arsenite along with other common mechanisms, thus explaining the phenomenon of cross-resistance.

**(4) SbIII, and SbV-Selected Mutants**

The use of SbIII that is not the prescribed form in clinical practice but represents the biologically active form of the drug would lead to the selection of resistant strains with different mechanisms and targets that are not necessarily involved in clinical isolates resistance since these are first exposed to SbV. However, cross-resistance between SbV, SbIII, and AsIII that was observed in some *Leishmania* species such as *L. tarentolae*, *L. major*, *L. Mexicana,* and *L. infantum* [15, 35, 36] allowed performing resistance experiments with SbIII. However, in some *L. donovani *strains, no cross-resistance was found [25]. Experiments on *L. panamensis *strains from clinical relapse showed that while amastigotes were SbV resistant, and promastigotes were sensitive to SbIII [37].

### 2.2. Phenotypic Analysis of Resistance

#### 2.2.1. Animal Model

The infection of mice or hamsters, which was among the first approaches for studying the sensitivity to antimonials *in vivo*, still faces the problem of variability related to the host and genetic background of these vertebrate hosts may have a major influence upon evolution after infection. They are currently mainly performed for immunological or genetic studies [31, 38, 39].

#### 2.2.2. *In Vitro* Macrophages Infection

Macrophages cultivation and their infection with promastigotes are useful tools for analysing the sensitivity of amastigotes. This technique is time consuming and it suffers from the variability of infectivity due to the growth characteristics of the strain and to *in vitro* metacyclogenesis level [40]. However, it remains a reference technique for studying the resitance phenotype of clinical isolates. Different cell lines can be used such as murine peritoneal exudate macrophages, murine bone marrow-derived macrophages, human monocyte-derived macrophages, and the human monocytic THP-1 cell line. Recently, it has been shown that either the infection kinetics or the activity of the drug especially antimonials depends on the host cell type. Consequently, macrophage population must be taken into account before any comparison between studies and there is a need for standardization [41].

#### 2.2.3. Axenic Amastigotes

The difficulty of sensitivity tests on infected macrophages is partially overcome by the axenic amastigotes approach, also called amastigotes-like. These stages are obtained by gradual changes of temperature and pH of cultured promastigotes until the parasites resemble amastigotes and express specific proteins and morphology of this stage [42]. Nevertheless, amastigote-like cells are heterogeneous and do not acquire all the characteristics of *in vivo* amastigotes. Indeed ultrastructural studies indicated that many amastigotes-like cells exhibit promastigote features [43]. Moreover, long-term culture may lead to reappearance of promastigote-like pattern [44]. While several studies have used this model and highlighted axenic amastigotes susceptibility to SbV [25, 35], other reports showed that pentavalent antimony is inactive on these forms and recommend intracellular amastigotes as the gold standard for *in vitro* sensitivity studies [42].

#### 2.2.4. Correlation to Clinical Outcome

It is important to assess whether *in vitro* tests used to determine the sensitivity of a clinical isolate reflect the response to treatment and clinical resistance. Faraut-Gambarelli et al. evidenced that in clinical isolates, among both HIV and immunocompetent patients, the IC50 of amastigotes within macrophages was significantly higher in patients who have not improved, compared to those who have responded to treatment. Similarly, strains from patients previously treated exhibited higher IC50 than strains from untreated patients. There was no difference in the IC50 for patients cured compared to those who developed a relapse after an initial improvement but iterative treatment with antimonials induced an increase in IC50 in strains isolated successively from the same patient. Thus, *in vitro* susceptibility of clinical isolates correlated well with clinical outcome in immediate treatment. In contrast, the long-term treatment did not appear correlated with *in vitro* tests since relapses occurred even in patients harboring susceptible strains [9]. Correlation between the clinical outcome and *in vitro* susceptibility tests was also observed in Indian field isolates [6]. A correlation was also found between treatment failure in patients, the IC50 of *L. donovani* promastigotes and the lack of response in the animal model suggesting the use of the IC50 study as a reliable test for resistance [45]. In an area highly endemic for visceral leishmaniasis in India, a double-blinded survey was performed in which intracellular amastigotes IC50 was determined for the first group of patients, whereas in the second group, patients were treated without testing the parasite sensitivity. Results showed a statistically significant improvement of cure rate reaching 86% in the first group compared to 35% in patients treated with SbV without *in vitro* tests [46].

Nevertheless, clinical outcome and *in vitro* susceptibility tests can be contradictory as shown for *L. donovani* clinical isolates tested for their sensitivity to SbV and SbIII. Indeed, Rijal et al. identified three profiles of resistance: strains sensitive to both forms of antimony, SbV resistant strains but SbIII sensitive and finally strains refractory to the two compounds. All patients unresponsive to treatment were infected with strains resistant to pentavalent antimony but this phenotype was also found in patients who responded to treatment. Some strains harvested from cured patients displayed *in vitro* resistance to SbIII. Finally, out of 3 patients whose strains were resistant to both SbV and SbIII, only one experienced treatment failure [47]. The same discrepancies were observed in a study performed on new world clinical strains [48]. These surveys suffer most from a lack of standardization regarding the criteria for treatment failure, the difference of resistance threshold, and the patients' population under study.

### 2.3. Molecular Determination of Resistance: Main Techniques

As mentioned above, these tests are carried out on selected *in vitro* mutants, on clinical human isolates, or on strains affecting other vertebrates such as *L. tarentolae*, a species widely used *in vitro*. The two parasite stages are examined but mostly promastigotes. Mutants are selected by exposure of parasites to increasing concentrations of the drug. Revertant strains are obtained by successive passages in culture of the resistant strain without drug exposure.

#### 2.3.1. Gene Amplification in *Leishmania*

Gene amplification was the first molecular resistance mechanism identified in *L. major* resistant to methotrexate (MTX) and displayed by the presence of H-circle [49]. The presence of circular amplicons in *Leishmania* is often the consequence of homologous recombination between repeated sequences. These events seem to be frequent in *Leishmania* and early associated to resistance phenotype. The presence of these elements has also been found in strains selected for other molecules unrelated to MTX as not only primaquine, and terbinafine, vinblastine but also arsenicals [50, 51]. This amplification may concern preexistant or novel H-circles [52, 53]. Their presence has been documented for wild strains of *L. major* and *L. tarentolae*. In addition to multidrug-related protein A (MRPA), two other genes have been identified within the H-circles: pteridine reductase 1 (PTR1) and H region terbinafine-associated resistance gene (HTBF) [54, 55].

Historical techniques for DNA and RNA analysis (Southern and Northern blotting) are now replaced by quantitative real time PCR (qPCR) or Reverse Transcription qPCR (RT-qPCR). qPCR is performed in order to compare amplification rate of a target gene between susceptible and resistant *Leishmania* strains. Results are expressed as threshold cycle (CT) and need to be standardized by using a housekeeping gene in the studied strains hence revealing possible gene amplification in resistant strain compared with wild type one [56]. Gene amplification can also be established by Southern blot by hybridization of gene of interest with its complementary probe [57, 58]. Quantitative reverse transcription PCR (qRT-PCR) is an approach used in this context to highlight gene overexpression of RNA which can be independent of amplification [59].

Recent advances in genomic approaches of *Leishmania* resistance were brought by DNA microarray technique concomitant with whole genome sequencing of *L. infantum* and *L. major* enabling an exhaustive prospection of differences between resistant and susceptible strains at gene level. Differential expression among the two strains is easily recognized by disposing of cDNA genomic bank and different genetic markers. DNA microarray technology allows testing a large set of genes at the DNA or RNA levels in a unique experiment and so is useful as screening test [60].

#### 2.3.2. Functional Cloning

Functional cloning of cosmid bank is a widely used technique for determination of genes functions. Cosmids harbouring a resistance marker such as hygromycine are transfected in *Leishmania*. This marker allows the selection of strains hosting the cosmid. Afterwards parasites are exposed to the investigated drug, such as antimonials [61]. There are two approaches for functional cloning depending on expected effect, dominant positive one and dominant negative one. In case of dominant positive cloning, the cosmid is transfected into a wild-type strain, and acquisition of a new function such as drug resistance is analyzed. By contrast in dominant negative selection resistant strains are transfected with the cosmid in order to obtain a reversal of resistance [62]. Once identified, cosmid of interest is retransfected in the strain under study to confirm the phenotype. Subsequently, the cosmid is sequenced and blasted with genomic bank in order to identify the resistance gene. In addition, this gene could be transfected into wild type strain to ascertain its role in resistance.

#### 2.3.3. Transfection and Deletion Experiments

Gene transfection is used to identify a new phenotype or a new functionality such as resistance. The strain transfected with gene of interest is compared to the same strain transfected with vector only, so keeping the original phenotype [63]. Protein localization can also be determined by cotransfection experiments with green fluorescent protein (GFP) [64].

Phenotype deletion at genomic scale is carried out by gene knockout (KO) approach. As *Leishmania* is a diploïd organism, two strategies are possible: a single allele KO resulting in a decrease in targeted functionality [61] and double knockout leading to phenotype suppression. This technique is uncommon because of the difficulty to obtain a double KO.

#### 2.3.4. Gene Polymorphism and Resistance

Isolates of *L. donovani* have been studied on the basis of their genetic diversities, variations, and molecular phylogenetic structure using fingerprinting approach-amplified fragment length polymorphism (AFLP) and identified a polymorphism percentage of 55%. Specific markers have been identified showing a different expression in resistant *L. donovani* strains [65].

Genetic studies, analyzing the whole genome, would present the advantage of searching gene of interest without any previous choice concerning their function. These studies need a very good definition of phenotype to lead to significant results.

#### 2.3.5. Pulse Field Gel Electrophoresis

Pulse Field Gel Electrophoresis (PFGE) can highlight differences at chromosomal scale between two strains in addition to the possibility of differentiation between linear and supercoiled DNA by varying pulse time [57].

### 2.4. Protein Level

Proteomic approach was initially reductive but last years global techniques as two-dimensional electrophoresis and more recently mass spectrometry offer a global analysis of cell proteome.

#### 2.4.1. Immunoblotting

Proteomic approach can be performed by immuno-enzymatic assays such as Western blot (WB), a semiquantitative technique that can reveal the presence of a protein and compare its relative abundance to other strains based on the band intensity [57]. Necessity of renaturation of protein during tranfert and availability of specific antibodies constitute the main limiting factors.

#### 2.4.2. Flow Cytometry

Flow cytometry was used in two aims: as a tools for *Leishmania* quantification in the last stage of sensitivity testing of infected cells and more interestingly for functional analysis of drugs transporter by using fluorescent drug analogs and specific inhibitors [31]. Specific inhibitors are commonly used for two purposes: the first is *in vitro* study of inhibiting an enzyme supposed to participate in resistance phenotype; the second is withdrawal of resistance profile in animal model using such inhibitor in combination with antimonials.

#### 2.4.3. Mass Spectrometry

This technique took an important place in recent years in proteomic studies. Its field of interest can range from screening of proteins giving an overall assessment of the differences that may exist between two strains to the sequencing of a protein. This approach is usually preceded by a two-dimensional electrophoresis or by liquid chromatography [66].

### 2.5. Metabolites Determination

Several techniques are used to study drugs uptake and accumulation in *Leishmania*. They are also utilized to determine concentration of metabolites involved in drug detoxification.

#### 2.5.1. High-performance Liquid Chromaography

High-performance liquid chromaography (HPLC) was mainly used for isolation, identification, and quantification of drug and/or thiols metabolites [57, 63].

#### 2.5.2. Injection-Coupled Plasma Mass Spectrometry

Injection-coupled plasma mass spectrometry (ICPMS) is used in *Leishmania* resistance surveys to measure differences in uptake and accumulation of SbV or SbIII depending on strain sensitivity or its stage [67].

**Figure 1:**
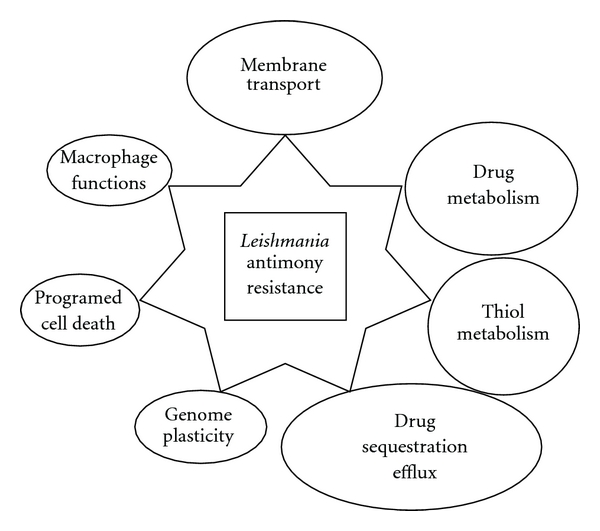
Main pathways involved in *Leishmania* resistance.

All these techniques allowed to discover and investigate one or several mechanisms related to *Leishmania* drug resistance. They involve drug entry, thiol metabolism pathway, drug transport and sequestration, programmed cell death and other minor mechanisms or cofactors as illustrated in Figure 1.

## 3. *Leishmania* Antimony Resistance Is Multifactorial and Involves Numerous Pathways

Resistance pathways in *Leishmania* share some common features with other microorganisms such as drug entry, drug metabolism, efflux and/or sequestration, programmed cell death along with action on host cell.

### 3.1. Drug Entry

The route of entry of antimonials is still unclear and does not appear to be the same for SbV and SbIII. Accumulation of SbV and SbIII by antimonials sensitive and resistant strains of different *Leishmania *species showed that both axenic amastigotes and promastigotes accumulated both forms of antimony using different carriers [67, 68]. SbIII entry is in part dependent on a parasitic plasma membrane protein, aquaglyceroporine (AQP1), also involved in the entry of other metbolites. AQP1 cloning and transfection in *L. infantum*, *L. major, *and *L. tarentolae* promastigotes expressing luciferase gene and infecting THP1 cell line showed increment of their susceptibility to SbIII and AsIII. A regain in susceptibility was noticed after transfection of AQP1 to *in vitro* selected mutants for SbIII resistance. These strains showed faster incorporation of SbIII and AsIII with 20- to 50-fold higher steady state accumulation amount compared to those transfected with the vector only. Amastigotes harvested from a resistant *L. donovani* field isolate recovered susceptibility after being transfected. Single allele knock-out in a *L. major *strain had led to a 10-fold decrease in its susceptibility, highlighting the significance of AQP1 amount in resistance phenotype [61]. There was no difference in AQP1 gene copy number between resistant and susceptible strains. In strains exhibiting decreased susceptibility to SbIII, this protein was underexpressed and a correlation was found between accumulation rate of SbIII and RNA expression level of AQP1 [62]. The AQP1 gene was found to be underexpressed in antimony resistant clinical isolates from Nepal in comparison with susceptible ones [69]. The composition of the amino acid sequence of AQP1 appears to influence the incorporation rate of SbIII in *Leishmania*. In fact, the replacement of alanine at position 163 by serine or threonine induced the decrease of the entry of the active form of antimony [70]. Other mutations in the sequence of this protein such as an alteration at Glu152 or Arg230 were associated with reducing SbIII entry [71]. These mutations were not found in resistant Indian clinical isolates with an underexpression of AQP1 [72]. Consequently, this mechanism is probably the first barrier that *Leishmania* involves in order to counteract the action of antimonials and induce resistance.

### 3.2. Drug Metabolism

**Figure 2:**
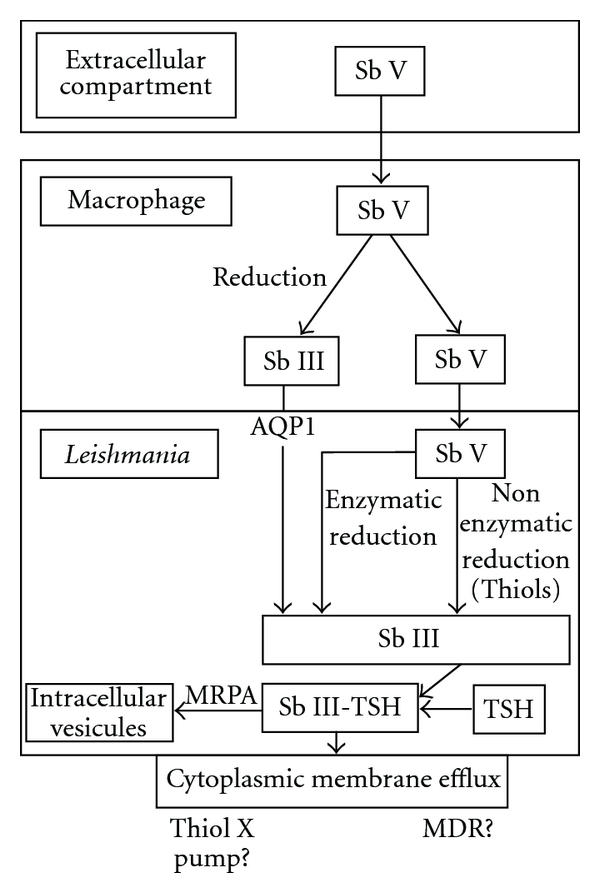
Antimony metabolism and transport inside macrophage and *Leishmania.* SbV: pentavalent antimony, SbIII: trivalent antimony, AQP1: acquaglyceroporine 1, TSH: trypanothione, MRPA: multidrug related protein A, MDR: multidrug resistance.

It is generally accepted that to be active on the parasite, the antimonial pentavalent form (SbV) has to be reduced to its highly active trivalent one (SbIII) [22, 35, 73–75]. The site of this reduction and its exact mechanism remain somewhat controversial but thought to take place in both macrophage and parasite. Major mechanisms are presented in Figure 2.

#### 3.2.1. Macrophage-Dependent Reduction

Evidence of macrophage involvement in reduction is that axenic amastigotes are less susceptible to Pentostam^R^ than intramacrophagic ones [35]. Furthermore, glutathione (GSH) which is the major thiol of the host cell can reduce SbV at 37°C. However the efficiency of this reduction decreases when pH increases from 3 to 8. In fact, at pH5, a condition encountered in phagolysosome, this reaction is more important than at pH7.2 (cytosol) and a little rate of SbV conversion is found although the highest amount of GSH is cytosolic [76]. Hence, it appears from these *in vitro* experiments that reduction does occur in macrophage but mostly in its acidic compartments such as endosomes, lysosomes, and phagolysosome. Other macrophage cytosolic thiols such as cystein and cysteinyl-glycin can also reduce SbV at rates even higher than GSH either in acidic or neutral pH conditions [77]. In a recent study, involvement of macrophages in SbV reduction was assessed in the human macrophage cell strain MM6. It has also demonstrated that cellular amount of SbIII is related to that of SbV. The latter seems to be in relation with extracellular SbV [78].

#### 3.2.2. Intraparasitic Reduction

The sensitivity of *Leishmania* depends on its ability to reduce SbV since antimonial reduction activity of resistant *L. donovani* amastigotes was impaired [68]. Reduction can be either enzymatic or nonenzymatic.

**(1) Enzymatic Reduction**

Two parasitic enzymes have been identified as being involved in SbV reduction, a thiol dependent reductase 1 (TDR1), and another one, called LmACR2.

The sequence of TDR1 was identified in *L. major *by *in silico* analysis, cloned, and the recombinant protein showed high conversion rate of SbV to SbIII using GSH as reducing agent. This enzyme was isolated from both stages of *L. major* and 10 times higher level of TDR1 was recovered from amastigotes than from promastigotes. Furthermore its activity is noticeably greater than nonenzymatic reduction [79]. LmACR2, an enzyme identified within *L. major* genomic sequence was cloned and transfected into *L. infantum* promastigotes: the derived amastigotes (infecting THP1 macrophages) were more susceptible to Pentostam compared to non transfected strain. Glutathione is also required for LmACR2 activity. Transfection of this protein in an Indian clinical isolate (*L. donovani*) induced sensitization of this strain to a lower dose of Pentostam compared to untransfected one [63].

**(2) Nonenzymatic Reduction**

This pathway involves parasite thiols which are represented by cysteine, spermidine, GSH, and trypanothione (TSH), the latter corresponds to the major thiol of the parasite [80]. *In vitro* experiments indicated that the SbV reducing activity of TSH was more relevant at pH7 than at pH5 and in all cases faster than SbV reduction with GSH [77]. Trypanothione results from the combination of GSH and spermidin [80]. This metabolic route involves two key enzymes, ornithine decarboxylase (ODC) and gammaglutamylcystein synthase (GCS). Production of thiols is crucial for the parasite in order to maintain a reduced environment inside the cell that counters the effects of oxidative compounds such as antimonials. In fact, the exposure of the parasite to heavy metals can lead to an impairment of its redox potential causing an efflux of intracellular thiols, the accumulation of intraparasitic disulfite, and the inhibition of two enzymes: trypanothione reductase and glutathione synthetase [81, 82]. Through conjugation to SbIII, trypanothione intends preventing antimonials action. It can so be inferred that enhancement of this machinery may result in antimony resistance. In fact, in *L. tarentolae* mutants selected for AsIII resistance and shown to be cross resistant to SbIII and SbV, thiols levels were higher than in wild type strain mainly for TSH [33].

Molecular approaches pointed out an amplification of the GSH gene within a linear amplicon described *in L. tarentolae, *selected for AsIII or SbIII resistance [17, 83]. Amplification and overexpression of GSH are not always correlated as shown for *in vitro L. tarentolae* selected for SbIII resistance; GSH1 and GSH2 were overexpressed but no amplification was found for GSH2 and it was discrete for GSH1 [83]. GSH1 overexpression was also found in clinical isolates [57]. In *L. tarentolae *revertant strain, a drop of GSH1 amplification was associated to a significant decrease in thiol levels without reaching the basal one. Transfection experiments carried out in this revertant strain with GSH gene resume resistance profile unlike wild type *L. tarentolae* which remains susceptible despite displaying an increase in thiols level that can be greater than that in resistant strains to AsIII [17]. Therefore, the intracellular TSH level is crucial to confer resistance pattern but is not sufficient alone to provide it. The level of thiols measured in *L. donovani *promastigotes was significantly higher in resistant strains with increased metabolic turnover and faster regeneration of thiols in order to counteract the production of ROS by antimonials. This effect is abolished by H_2_O_2_ which causes a depletion of thiols thus restoring the efficiency of antimony [84].

For ODC, an overexpression without amplification was found in both *L. tarentolae* AsIII resistant and revertant strains and for *in vitro* selected *L. tropica* and *L. mexicana*. Since there was not gene rearrangement nor increased translation, this overexpression in the absence of amplification may be the consequence of an enhanced RNA stability [85]. In clinical strains, ODC gene was found amplified; this amplicon was not extrachromosomal and protein amount was increased [57]. In clinical isolates, ODC overexpression was found in resistant strains of *L. braziliensis* and was associated with GSH overexpression in *L. donovani *[57, 86].

Specific inhibition of GSH and ODC with buthionine sulfoximine (BSO) and difluoromethylornithine (DFMO), respectively, further supported their role in resistance. This suppression resulted in a partial reversal of resistance in resistant strains and in a decrease in TSH eventhough remaining at residual level higher than in wild type strains [16, 32, 37, 85]. Reversal of resistance was also obtained by BSO in unresponsive *L. donovani *and *L. tropica* clinical isolates [5, 39].

Inhibition of GSH synthesis by BSO was also tested *in vivo* in mice infected with resistant and susceptible strains of *L. donovani*. The combined treatment SbV + BSO significantly increased sensitivity and decreased resistance, respectively [39]. However, BSO had no effect on *L. panamensis* clinical strains resistant to SbV [37].

As reported above, intracellular glutathione is crucial for cell survival by maintaining a redox balance and protection against toxic agents that cause oxidative or chemical stress. Impairment of production of GSH is toxic to cells and its inhibition has been successfully used to increase the sensitivity of number of intracellular pathogens and cancer cells to different drugs. Infection of mice with SbV sensitive *L. donovani* strains is associated with increased macrophage GCS mRNA unlike resistant strains which induce a reduction in intramacrophagic expression of GCS which leads to a loss of GSH and generates an oxidative environment into the host cell. Consequently, the reduction of SbV to its highly active SbIII is diminished in macrophage. In contrast, these same resistant strains display an overexpression of GCS compared to susceptible ones leading to a higher rate of thiols in the parasite in order to compensate an oxidizing environment generated in macrophage [87].

In *L. infantum* axenic amastigotes selected for SbIII, SAHH gene, which is responsible for the conversion of S adenosyl homocystein to homocystein, the cystein precursor was overexpressed. Interestingly, cystein which is a glutathione precursor was the only thiol with increased concentration in this study. SAHH was also amplified in SbIII resistant *L. tarentolae *[83].

### 3.3. Drug Transport

In several microorganisms, membranous transport plays a major role in drug resistance. ATP Binding Cassette (ABC) family is a set of proteins involved in resistance either by drug efflux or by its sequestration. This efflux pumps system was first described in tumor cells, and the derived phenotype was named multidrug resistance (MDR) resulting in refractoriness to chemotherapy. In *Leishmania*, 8 subfamilies of ABC transporters genes, present among 19 different chromosomes, were identified when *L. major *whole genome was compared with established ABC proteins sequences [59]. Among these, two subclasses of ABC transporters seem to be involved in *Leishmania* resistance:

1. carriers with high similarity to P-glycoproteins in mammals that confer an MDR phenotype similar to that seen in cancer cells,
2. homologous to the Multidrug Resistance-related Protein (MRP) in humans [88].

#### 3.3.1. MDR

MDR profile was found in resistant strains of *Leishmania *and the MDR1 gene was amplified in mutants selected for resistance to vinblastine, daunomycin, or to puromycin [89–92]. MDR-class transporters are involved in extrusion of hydrophobic molecules but not hydrophilic ones as SbIII. Indeed, this effect has not been demonstrated for antimonials.

#### 3.3.2. MRPA

MRPA formerly called PGPA for P-glycoprotein A is the most studied ABC transporter in *Leishmania* and its role in resistance is well documented *in vitro* [93]. MRPA gene is often located on an amplified H-circle extrachromosomal DNA [21, 83, 88, 93–95] which initially belongs to a locus in chromosome 23. This locus is flanked at each side by repeated sequences of 1389 bp that present a sequence homology of 100% in a *L. infantum* strain selected for SbIII resistance [60]. These repeats were also found on the same chromosome of *L. major *and *L. braziliensis *[96]. However, in *L. mexicana* selected for AsIII resistance, it was located in a linear amplicon [95]. MRPA gene is frequently amplified/overexpressed in strains resistant to heavy metals [60, 83]. The level of resistance is associated with this amplification as demonstrated by a comparative study between *in vitro* selected *L. major* and *L. braziliensis* strains to antimonials [96]. In fact, resistance was associated to H-circle amplification in *L. major* but not in *L. braziliensis *and the latter remained significantly more sensitive to antimony. Similarly, decreased amplification was observed in revertant strains. Initially susceptible strains transfected with MRPA gene acquire a certain level of resistance to SbIII or AsIII but this level is variable depending on species even if equal band intensity can be found in WB for MRPA recombinant protein [95, 97, 98]. This variability can be explained by an interspecies difference in basal level of thiols. Evidence of combined action between MRPA and thiols in heavy metals resistance was confirmed by cotransfection experiments of MRPA and GSH genes which confer resistance in wild type *L. tarentolae* and restore a high resistance level in revertant strain unlike GSH transfection alone [17]. Unlike classical ABC proteins, MRPA does not act by efflux at the plasma membrane. In fact, this protein is located in an intracellular compartment close to the flagellar pocket of the parasite recognized as the site of endocytosis and exocytosis and acts by the sequestration of the SbIII-TSH complex since free SbIII or AsIII is not transported by MRPA [62, 78]. Therefore, thiols act by two opposite mechanisms: on one hand, they are involved in drug activation and on the other, in its inactivation via its sequestration. In parallel with drug sequestration, MRPA amplification seems to cause a decrease in drug entry rather than increasing its efflux [99]. This can be explained by a possible dominant negative role played by MRPA on membrane proteins. Plasma membrane efflux mechanism was demonstrated in *Leishmania *in membrane-enriched everted vesicles of *L. tarentolae* in which an ATP calcium-dependent protein was involved in AsIII-GSH transport not mediated by MRPA. In fact, double knock-out of MRPA gene did not alter this transport which was inhibited to some extent by Pentostam indicating competition between the two related metals. In addition, the activity of this transporter is not increased in membranes from mutants that overexpress MRPA suggesting that this thiol X efflux pump is encoded by another gene. Nevertheless, no protein was yet identified [100].

Functional studies of the ABC family pumps were performed on clinical isolates of *L. donovani* (promastigotes and axenic amastigotes) using rhodamine123 (R123) as substrate for MDR, calcein for MRP, and by employing specific inhibitors. The R123 accumulation was found greater in resistant strains and its rate was not modified by verapamil, a specific inhibitor of MDR, suggesting the absence of a classical MDR activity. The accumulation of calcein was lower among these resistant strains for both promastigotes and axenic amastigotes. Use of probenecid, an inhibitor of MRP, did not lead to a significant increase in calcein unlike ATP depletion indicating the existence of an MRP-like pump more active in resistant isolates [101]. The role of MRPA and the level of resistance depend on the rate of residual SbIII as demonstrated in a study that initially showed no correlation between the *in vivo* efficacy of SbV and the residual SbIII in parasite but highlighted the importance of this residual rate for the action of MRPA. Indeed, although strains that overexpress MRPA were all resistant to SbIII, cross-resistance to SbV was observed only in strains that exhibited a high SbIII residual rate, indicating that this refractoriness was directed against the antimony trivalent form [102]. Leprohon et al. found that MRPA was overexpressed in SbIII resistant *L. infantum,* compared to wild type by microarray approach. Interestingly, two other proteins of the ABC family were overexpressed, namely, ABCA3 and ABCH1. Identical results were obtained in another independently selected strain. However, in wild-type strains, only MRPA transfection confers SbIII resistance [59]. Implication of ABCA3 in vesicular trafficking and exocytosis pathway supports an indirect implication in *Leishmania* resistance [103].

All these proteins were located in intracellular compartments. Their role in resistance to antimonials was investigated in a revertant strain of *L. tarentolae*. Only transfections of ABCC4-GFP and ABCC5-GFP were accompanied with a two-fold increase of resistance level [64].

#### 3.3.3. Other ATP-Dependant Transporters

Other proteins of the MRP subfamily of ABC have been identified as PGP B, C, D, and E. However, single transfection of their genes does not confer resistance [88]. The role and action of these proteins remain unknown, but their sequence similarity with MRPA indicates that they may correspond to membrane transporters involved in antimonials efflux. Recently, a second protein belonging to the ABC family transporters, and named PRP1, was identified by cloning after selection by pentamidine. This protein confers cross-resistance to antimonials [108].

Some genes identified *in vitro* as involved in resistance to antimonials were investigated in clinical isolates from Sudan and France [56]. MDR1 was amplified in 65% of isolates and was the only statistically associated with *L. infantum*. Amplification of MRPA in concomitance with GSC was observed in some strains confirming the joint action of thiols metabolism and sequestration of conjugated antimony. This amplification is stable in successive samples for the same patient. Nevertheless, it was not possible to establish a correlation between therapeutic response and gene amplification observed in this survey since the French patients were treated with liposomal amphotericin B while the clinical data were lacking for Sudanese isolates. However, it supported the presence of an amplification of some genes identified *in vitro* as taking part in resistance. The singularity of this study is the presence of an amplification of the MDR1 gene which is found amplified in *in vitro* strains selected for resistance to molecules other than heavy metals. The significance of this issue remains difficult to elucidate and we can only speculate on the role of MDR1 in antimony resistance in clinical isolates. This phenomenon can be explained by adaptative functions of the parasites when exposed *in vivo* to drugs used for treatment of another disease. This drug can select in *Leishmania* a new function, such as MDR1 amplification in this case, without any linkage to antimony therapy. DHFR gene is another illustration of this phenomenon. This gene is related to folates pathway and antifolates are used in several affections such as malaria where DHFR is amplified in *Plasmodium* resistant strains [109]. Although antifolates are not used in *Leishmania* treatment, amplification of DHFR gene occurs *in vitro* after methotrexate selection and in clinical strains [56, 83]. In other situations, selection by other molecules can lead to cross-resistance. Indeed, miltefosine, a drug recently introduced in antileishmanial therapy, has plasma membrane as targets [110] and it was suggested that antimony resistant *Leishmania *also shows membrane modifications [111]. Combined with antimonials wide use in some endemic areas, this mode of action led to cross-resistance between these two molecules [112].  Table 1 summarizes the most important molecular targets identified either in *in vitro* selected mutants or in field isolates of *Leishmania*.

### 3.4. Programmed Cell Death

A comparative proteomic analysis between two *L. donovani *Indian clinical isolates resistant and susceptible to SbV [66] identified proteins having already known roles in programmed cell death (PCD) as the 14-3-3 protein present in higher quantities in the resistant strain, and which belongs to a family of conserved proteins able to bind other phosphorylated proteins involved in apoptosis [113]. Heat Shock Proteins (HSPs) are also involved in PCD by modulating some steps in the apoptosis pathway [114]. In fact, Antimonials induce the production of a variety of HSPs (HSP70, HSP81, and HSP65) and their role is supposed to be protective against the toxic effect of these drugs. HSP70 and HSP83 were overexpressed. Transfection of a cosmid library in *Leishmania* parasites and their selection with SbIII identified in resistant strains a cosmid containing HSP70 and HSC70. The protein expression of HSP70 increased when concentration of SbIII was incremented. Overexpression of HSP70 was found in revertant strains suggesting its stability. However, transfection experiments of HSP70 or HSC genes did not give rise to any resistance thus assuming a minor role in resistance. Similarly, Brochu et al. showed that level of HSPs of different molecular weights is increased in the presence of arsenicals. However, transfection of HSP gene does not confer resistance directly but rather increases parasite tolerance to heavy metals [115]. Small kinetoplastid calpain-related protein 1.14 (SKCRP1.14), which was underexpressed in *L. donovani* resistant strain [90], is often associated to other proteins identified as effectors of PCD in kinetoplastidae [116]. Thus, it is conceivable that the process of apoptosis in resistant strains is impaired. This is corroborated by evidence of reduced DNA fragmentation and formation of bridges in resistant strains compared to susceptible ones.

### 3.5. Other Mechanisms and Cofactors of Resistance

Tryparedoxin peroxidase (Trper), an enzyme responsible for peroxides ions detoxification in *Leishmania,* also plays a role in antimonial resistance as shown in *L. donovani *promastigotes and amastigotes transfected with this protein. In fact, either *in vitro* selected strains or field isolates became less susceptible to SbIII [105, 106]. Likewise, the resistance profile was also demonstrated in resistant mutants of *L. tarentolae* strains by overexpression of the recombinant protein in initially susceptible strains [107].

Histones H4, H2A, and H1 showed differential expression pattern between susceptible and resistant strains of *L. donovani *clinical isolates [117]. Nevertheless, H2A gene transfection in *L. donovani *susceptible strains displayed no significant increase in the ED50 for SbIII. Differential expression also concerned MAPK, a mediator which plays an important role in intracellular multiplication of the parasite, the morphogenesis of the flagellum and consequently in the virulence of the parasite during host infection [117–119]. LinJ05_V3.0830 was 4 times overexpressed in *L. infantum* resistant strains selected for SbIII resistance. However, transfection experiments in sensitive and revertant strains indicated no significant change in sensitivity [60].

*Leishmania* synthetizes phosphoproteoglycans (PPGs) that are secreted or present at the parasite membrane in the two stages. These proteins play an important role in parasites survival [120]. Quantitative analysis highlighted greater amount of PPG in resistant strains.

Various other proteins were overexpressed in resistant clinical isolates of *L. donovani* at membrane and cytosolic levels such as GPI transaminase protein, a cysteine-rich leucine protein, a 60S ribosomal protein L23a, proliferative cell nuclear antigen (PCNA), the alpha5 subunit of the proteasome, carboxypeptidase, enolase, fructose-1,6-bisphosphate aldolase, and a beta tubulin chain [121]. In *L. infantum* axenic amastigotes, proteomic approach highlighted overexpression of arginosuccinate synthetase and an underexpression of kinetoplastidae membrane protein (KMP-11) with an expression level correlated with the resistance phenotype [122]. Other *L. infantum* strains overexpressing a protein which belongs to the superfamily of leucine-rich repeat (LRR) proteins, became resistant to SbIII for axenic amastigotes and to SbV for intramacrophagic ones [122].

### 3.6. *Leishmania* Resistant Strains Display Chromosomal Changes

In an *L. infantum* mutant strain selected for SbIII resistance, the expression of whole chromosomes was modulated, compared to the sensitive one [60]. Gene overexpression involved rather chromosomes 1, 11, and 25, in opposition with chromosome 9, 12, and 32 where genes were found underexpressed. Aneuploidy was also observed in resistant strains. The rate of aneuploidy correlated with the level of SbIII resistance. Interestingly, aneuploidy and the presence of the circular amplicon carrying MRPA gene disappeared in the revertant strain. However, haploidy for chromosomes 12 and 32 has persisted; this possibly explains the remaining of resistance at residual level.

## 4. Field Isolates Can Exhibit Resistance Pattern Different from *In Vitro* Mutant

In a study on resistant field isolates of *L. donovani*, increase of thiols level was much lower than that observed in mutant strains selected *in vitro* explaining in part the higher resistance observed in mutants compared with clinical unresponsive strains [45]. Trypanothione reductase gene was 2.5 times amplified in resistant isolates thus maintaining a high level in thiols and counteracting the effect of SbIII. However, GCS gene was not amplified like in *L. tropica* clinical strains [104]. Unlike *in vitro* selected mutants in which TSH level is usually increased, some clinical resistant strains display cystein and GSH overproduction but not TSH [57]. Amplification of MRPA can also occur in susceptible clinical strains [69]. As that of AQP1, GCS, and ODC genes were surprisingly underexpressed in resistant strains in intracellular amastigotes. This modulation was also observed for GSC in the same proportions in promastigotes; it was lower for AQP1 and absent in the case ODC. These findings are in opposition with most studies on *in vitro* resistant mutants and other surveys on clinical isolates. This difference can be attributed at least for the *in vitro* mutant strains to different mechanisms depending on the used antimonial form. Indeed, in this study it was observed that SbV exposure of amastigotes induced an underexpression of AQP1 involved in the entry of antimony in the parasite cell, but also inhibition of enzymatic and nonenzymatic pathways involved in pentavalent compound reduction. This mechanism has been described in axenic amastigotes of *L. donovani *[68]. The number of copies of AQP1 gene may not correlate with the level of expression as determined in *L. donovani *resistant clinical isolates, which displayed lowered SbIII accumulation, an underexpression of AQP1, but increased copy number of its gene [72]. Inconsistency between *in vitro* resistance tests and clinical outcome was also found in cutaneous leishmaniasis isolates from Peruvian subjects [123]. Some molecular targets were tested in promastigotes ofamerican cutaneous leishmaniasis clinical isolates (*L. braziliensis*, *L. guyanensis*). For *L. braziliensis* there was no difference in expression for MRPA, GSH, trypanothione reductase, and TDR1 in relation to treatment issue, while for *L. guyanensis*, GSC gene was overexpressed in strains that caused treatment failure [124].

Recently, an amplified sequence of 1254 Kb was found in *L. donovani* resistant field isolates [58]. Hybridization experiments have shown that this sequence did not match any gene of ABC family or with other genes involved in resistance. This sequence does not contain transmembrane domain eliminating a possible role in efflux. However, antibodies against the recombinant protein of this sequence allowed assessing its membrane localization. The functional study of this sequence confirmed the absence of an MDR-like pump that can be inhibited by verapamil or an MRP-like pump.

Another experiment using intramacrophagic amastigotes labeled with luciferase from susceptible and resistant *L. donovani* clinical isolates showed that refractory clinical strains to SbV displayed also *in vitro* resistance to this compound. This strain was also significantly more resistant to SbIII than the susceptible one that allowed performing experiences on promastigotes, easier to carry on. Genes usually involved in *in vitro* resistance as AQP1, MRPA, or GSH were not modulated in the resistant strain [66].

## 5. Conclusion

Antimonials remain the first line treatment of leishmaniasis in medical and veterinary fields and the emergence and spreading of resistance is still an important public health problem.

This phenomenon is complex and multifactorial. Experimental studies have pointed out the role of numerous mechanism involved in resistance. Field studies gave partial information and definition of the resistant phenotypes appeared to be difficult because of the influence of host factors and lack of exhaustivity.

*In vitro* sensitivity testing on infected cells remains the better global estimation of *Leishmania *resistance and substitutive, informative, molecular tests are needed. All these observations lead to a better understanding of resistance mechanisms in *Leishmania*. Progress is undeniable; however, the discrepancies between *in vitro* and clinical resistance remain numerous, even for surveys held for clinical isolates of the same species in adjacent geographic areas. This review illustrated the difficulties to identify markers of resistance that can be standardized with a test easy to implement, replacing the time consuming determination of IC50 in the amastigote-macrophage model. Additional work, focusing on search for reliable molecular markers, would be valuable for the community.

## Figures and Tables

**Table 1:** Relevant molecular targets involved in *Leishmania* antimony resistance.

	DNA amplification	RNA overexpression	RNA lowered expression	Functional cloning/ transfection	*In vitro* selected mutants	Field isolates	References
MRPA	++++	++++		+	yes	yes	[21, 56, 83, 88, 93–95, 97, 98]
GCS	+++	+++	+ [69]		yes	yes	[56, 57, 69, 83
ODC	++	+++	+ [69]			yes	[57, 69, 86
Tryr	+					yes	[104]
Trper				++	+	yes	[105–107]
AQP1			++++	+	yes	yes	[61, 62, 69, 72]
LmACR2				+	yes	yes	[63]

The number of “+” signs is relative to the importance of the mechanism. For GCS and ODC, RNA-lowered expression is found only in one survey.

MRPA: multidrug resistance related protein A.

GCS: gammaglutamylcystein synthase.

ODC: ornithine decarboxylase.

Tryr: trypanothione reductase.

Trper: tryparedoxin perxydase.

AQP1: aquaglyceroporin1.

LmACR2: Leishmania major antimony-arsenite reductase.
